# Larva migrans in BALB/c mice experimentally infected with *Toxocara cati* ensured by PCR assay

**DOI:** 10.1186/s12917-022-03366-6

**Published:** 2022-07-05

**Authors:** Majid Naderbandi, Mohammad Zibaei, Ali Haniloo, Farzaneh Firoozeh, Zahra Hatami, Elham Shokri, Kensuke Taira

**Affiliations:** 1 Department of Parasitology and Mycology, School of Medicine, Zanjan University of Medical Sciences, Zanjan, Iran; 2 Department of Parasitology and Mycology, School of Medicine, Alborz University of Medical Sciences, P.O. Box: 3149779453, Karaj, Iran; 3 Department of Microbiology, School of Medicine, Alborz University of Medical Sciences, Karaj, Iran; 4 Laboratory of Parasitology, School of Veterinary Medicine, Azabu University, Fuchinobe, Sagamihara, Kanagawa Japan

**Keywords:** *Toxocara cati*, Larva Migrans, BALB/c mice, Microscopic larvae count, PCR

## Abstract

**Background:**

*Toxocara cati*, the cat roundworm, is a parasitic nematode that known to cause toxocariasis in intermediate hosts and humans. In this study, we characterized the dynamics of *T. cati* larvae migration in BALB/c mice after inoculation with eggs and ensured the migration detecting the larval DNA by a PCR. To evaluate the dynamics of larval migration and distribution, twenty-four BALB/c mice were orally inoculated with 2500 T*. cati* infective eggs and the visceral organs of the infected animals were examined by pepsin digestion and microscopic parasite counts, followed by PCR at day 1 to 28 post-inoculation.

**Results:**

The PCR assays were successfully used for detection of *T. cati* larvae in tissue samples and *T. cati* larvae and the DNAs were found in the liver, lungs, heart, kidneys and the brain. We detected *T. cati* in 92.2% of tissue samples by PCR, 30% higher than the conventional pepsin digestion technique.

**Conclusion:**

Our findings demonstrated that the PCR assay is a sensitive and specific for the detection of *T. cati* larvae. Therefore, it could become a useful tool for the investigation of the dynamics of larval migration and *Toxocara* infection in murine model.

**Supplementary Information:**

The online version contains supplementary material available at 10.1186/s12917-022-03366-6.

## Background

*Toxocara cati*, a global cosmopolitan and typically neglected parasitic zoonosis, is an ascarid nematode in the family Toxocaridae [1, 2]. Felids are the most common definitive hosts of *T. cati*, while humans as one of the many mammalian and many animals such as rodents and birds can serve as paratenic hosts [3]. Small mammals such as mice accidentally acquire the infection when they ingest infective *Toxocara* species eggs and are considered as a natural source of infection in the environment for predators [4, 5]. On the other hand, humans become infected by ingestion of infective eggs from various environmental sources contaminated with definitive hosts feces. Toxocariasis encompasses four clinical syndromes: visceral larva migrans, ocular larva migrans, neurotoxocariasis, and hidden or covert toxocariasis. Presentation and outcome of infection are determined by interaction between the host and the parasite [6].

Experimentally, some animals were infected with helminthic parasites and encapsulated larvae were found in the tissues of internal organs of these animals. BALB/c mice has been used as a model to study the host-parasite relationship in human and/or animal toxocariasis [7–9]. The larvae hatch and migrate out of the intestine to become lodged in organs and tissue [8]. Infection of rodents with *T. cati* has been carried out in different strains of rats and mice [5, 10]. However, the kinetic time-lines and migratory route have not been established in the BALB/c strain. The gold standard for the diagnosis of the majority of helminthic infections is conventional microscopy and shown it is one of the most important diagnostic method to determine parasite migration routes and lesions caused by *Toxocara* species in paratenic hosts [5, 8, 11–15].

Polymerase chain reaction (PCR)-based techniques have revolutionized many areas of study including parasitic infection diagnosis. PCR is widely used mainly because its sensitivity allows the amplification of gDNA fragments from minute amounts of parasite material [16]. Performing a PCR assay does not require any parasitological proficiency and can be easily adapted by diagnostic laboratories that routinely use molecular assays to detect other pathogens. Several reports have shown a high PCR sensitivity for detection of *Toxocara* DNA in various biological samples from different hosts [17–19].

In the current study, the sensitivity between conventional methodology and PCR was compared to characterize *T. cati* infection in BALB/c mice. This comparison was carried out in two steps. First stage, initially was established the dynamics of infection by digestion method and microscopic observation, and PCR analysis of tissue samples. Then, the larvae migration route by PCR and parasitological examination was evaluated.

## Methods

### Ethical consideration

The study design, including its ethical aspects, was reviewed and approved by the Ethics Committee of Zanjan University of Medical Sciences (IR.ZUMS.REC.1400.133).

### Parasites

Worm samples were taken from cats with *Toxocara* referred to Karaj veterinary clinics by taking anti-helminthic drugs (with the consent of animal owners). Unembryonated *T. cati* eggs were collected from the faeces of naturally infected cats and harvested from adult female worms, incubated for three weeks at 25 °C for embryonation, following the technique described by Zibaei and Uga, [20]. Before inoculation, the eggs were washed three times with distilled water to remove formalin.

### Animals

Specific pathogen-free male BALB/c mice aged 6–8 weeks, weighing 18–22 g, were used in the study. Animals were purchased from a commercial experimental animals company (Royan Animal Breeding Center, Karaj, Iran). Similar to earlier research, mice were individually housed in plastic cages in an animal maintenance room for the duration of the experiments [5, 12]. The room was maintained at approximately 20–24 °C and 50 ± 5% relative humidity, and operated on a 12 h light/dark photoperiod. Water and pelleted commercial food were supplied ad libitum, and cages were cleaned on a regular basis. The animal care and experimentation were performed according to the national guidelines and protocols approved by the Research Ethics Committee of Alborz University of Medical Sciences in accordance with the National Institute of Health Guide for the Care and Use of Laboratory Animals (NIH Publication No.85–23, revised 1996).

### Experimental design and protocol

Twenty-four animals were divided into 7 groups: control group (*n* = 6, with distilled water), six infected groups (*n* = 3/group). Mice were allowed to acclimatize for 1 week prior to commencing experiments. Total, 18 mice of infected groups were each orally inoculated with 2500 T*. cati* embryonated eggs suspended in 0.5 mL of saline solution, by stomach tube. The animals were monitored daily for clinical signs during routine animal care.

### Recovery of larvae

**Fig. 1:**
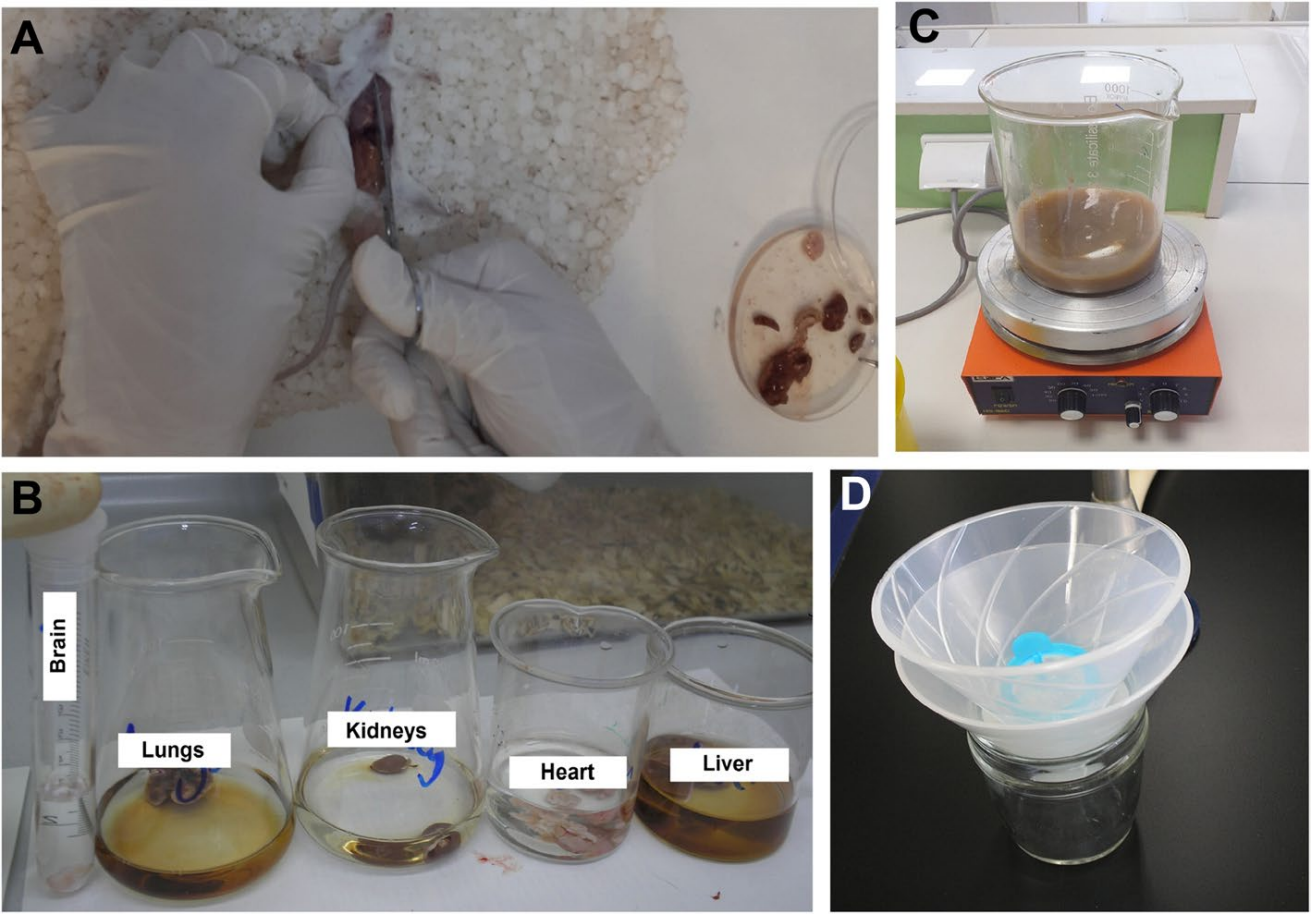
Pepsin digestion method. **A** Anesthesia and euthanization of BALB/c mice using the standard laboratory methods, (**B**) Tissue digestion by digestive solution (pepsin, HCl, and water), (**C**) Complete digestion by magnetic stirrer, and (**D**) The modified Baermann technique for the recovery and counting of larvae

The infected animals were euthanized using Ketamine-Xylazine (60 mg/kg ketamine with 8 mg/kg xylazine) on days 1, 3, 7, 14, 21 and 28 post-inoculation. Larvae were recovered and counted from the liver, lungs, heart, and kidneys using a pepsin digestion method and the modified Baermann technique (Fig. 1) [5]. The brain were removed individually and pressed between two slide-glasses to count the larvae under a light microscope. The controls were euthanized at the end of the study days.

### Larval DNA

After necropsy, target organs including the liver, lungs, heart, kidneys and brain were removed in infected and control groups of the studied animals, as previously described [19]. For subsequent extraction of genomic DNA, these tissue samples were fragmented with a scissors and pointed forceps in 5 fractions of 10 mg to 20 mg. Tissue samples were frozen and stored at -80 ºC. After at least 24 h, total DNA was isolated from the stored samples using a commercially available kit.

### DNA isolation

Purify gDNA was isolated from samples using Favorprep™ Tissue Genomic DNA Extraction Mini Kit (Favorgen Biotech, Ping-Tung, Taiwan, and China) regarding the manufacturer’s instruction. The isolated DNA was eluted in 10 mM Tris–HCl and stored at -20 °C until further use.

### PCR primers

A primer pair JW4 and NC2 (Forward primer 5′-ACTGTCGAGGATGAGCGTGA-3′ and Reverse primer 5′-TTAGTTTCTTTTCCTCCGCT-3′, respectively) was used to target a 600-bp fragment of the ribosomal ITS1 and ITS2 regions of *T. cati* [21].

### PCR conditions

All PCR reactions were carried out in 25 μL final volume containing 12.5 μL of Super master mix 2X (Favorgen Biotech, Ping-Tung, Taiwan, China), 1.5 μL of each forward and reverse primer (10 pmol/μL), 3 μL of DNA template. The final reaction volume was adjusted to 25 μL with sterile demineralised water. The detailed PCR temperature cycling conditions as described previously [21] as follows: initial denaturation at 94 °C for 5 min; 35 cycles of denaturation at 94 °C for 30 s, annealing at 55 °C for 30 s, and elongation at 72 °C for 1 min. The final cycle was followed by extension at 72 °C for 7 min. The PCR products were separated by electrophoresis on a 1% agarose gel and visualized with a UV transilluminator (UV Transilluminator, QUANTUM SD4-1000, VILBER, France) after staining with 3 μg/mL GelRed.

### Statistics

Comparison of sensitivity between conventional and PCR techniques to detect larval infection was evaluated by the Chi-square test and the agreement between techniques, by the McNemar Test. The level of significance was set at 5% (*P*-values < 0.05). All data analyses were with SPSS 21.0 statistical software.

## Results

### Animals and dynamics of infection

The outcome of the infection was initially determined by counting the number of larvae that migrated to the tissue in the days after inoculation. The maximum number of tissues larvae was 40 (± 11.34) larvae observed on day 3 post-inoculation. From the 7th day onwards, the amount of recovered larvae steadily decreased until day 28.

Performing the PCR using the genus primer detected the *T. cati* DNA in samples from days 1 to 28, the last day of evaluation. Interestingly, the more intense parasite DNA amplification bands coincided with the highest amount of parasite larvae recovered with the digestion method.

### Migration route characterization by digested tissues analysis

This analysis was initiated on day 1 post-inoculation and at this time parasites were located in the liver tissues in 3 BALB/c, and at the heart in one BALB/c in the six groups each containing 3 animals. Larvae were not found in the kidneys and brain tissues 24 h after infection. However, appearance of larvae in the lungs was clear at 24 h. At this time, two of three animals already showed larvae located inside the tissue. The analysis at days 3 and 7 post-inoculation revealed that all animals had larvae in the liver, lungs, heart and kidneys samples. At this time, no larvae were found in any other tissues that were examined. At days 14, 21 and 28 post-inoculation, the larvae disappeared from the liver samples examined. It is interesting, at these times, some samples from the nine infected-animals had larvae inserted in the lungs, heart, and kidneys. On the other hand, no larvae were observed in the examined brain tissues on the mentioned days. The number of recovered larvae is documented in Table 1. There was no significant difference between larval recovery in the tissues and days post-inoculation (*P* > 0.05).

**Table 1:** Number of larvae and detection of DNA of *Toxocara cati* from BALB/c mice (*n* = 3) inoculated with 2500 eggs and necropsied at different days post-inoculation

DPI^a^	***T. cati* larvae**											
	**Liver**		**Lungs**		**Heart**		**Kidneys**		**Brain**		**Total**	
	**Number Mean**^b^ **(± SD)**	**DNA**^c^	**Number Mean (± SD)**	**DNA**	**Number Mean (± SD**)	**DNA**	**Number Mean (± SD)**	**DNA**	**Number Mean (± SD)**	**DNA**	**Number Mean (± SD)**	**DNA**
1	18 (± 4.00)	+	18 (± 5.30)	+	1 (± 0.58)	-	0	+	0	-	34 (± 9.68)	+
3	10 (± 1.15)	+	27 (± 4.00)	+	2 (± 0.57)	+	1 (± 0.58)	+	0	-	40 (± 11.34)	+
7	1 (± 0.58)	+	13 (± 2.52)	+	2 (± 1.55)	+	8 (± 1.53)	+	0	-	24 (± 5.54)	+
14	0	+	4 (± 1.15)	+	1 (± 0.58)	+	1 (± 0.58)	+	0	-	6 (± 1.73)	+
21	0	-	2 (± 0.57)	+	0	+	0	+	0	+	2 (± 0.89)	+
28	0	-	1 (± 0.58)	+	0	+	0	+	0	+	1 (± 0.45)	+

^a^Days post-inoculation

^b^Larvae were counted under a light microscope after a pepsin digestion method

^c^*Toxocara cati* DNA was amplified from 20 mg of tissue by PCR

### Tissue parasite detection by PCR

**Fig. 2:**
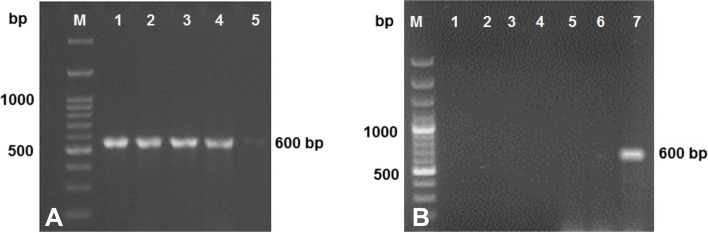
DNA amplification of *Toxocara cati* recovered from BALB/c mice by electrophoresis on 1% agarose gels in the analysis of PCR products. **A** Infected groups, lane M: 100 bp DNA size marker; lanes 1–5: 600 bp DNA from the liver, lungs, heart, kidneys, and brain respectively. Full-length gel image is provided in Supplementary Fig. 2A. **B**: Control group, lane M: 100 bp ladder; lanes 1–5: the tissue samples without DNA; lane 6: the negative control; and lane 7: DNA standard. Full-length gel image is presented in Supplementary Fig. 2B

The size of PCR product amplified from DNA samples from tissue specimens using the primers JW4 and NC2 were in size 600. This band was detected in DNA samples obtained from the different tissues of sixteen mice in groups of three animals that were euthanized 1 day after infection. This specific band was also amplified in DNA samples from the liver (seven animals), lungs (sixteen animals), heart (five animals), kidneys (four animals), and from the brain (two animals) at days 1, 3, 7, 14, 21, and 72 post-inoculation, respectively. This 600-bp specific amplification band was also present in brain DNA samples obtained from one animal at day 21 and from another BALB/c at the 28 day-period evaluation (Fig. 2A). As expected, this specific band was not detected in any of the samples obtained from the control experimental group (Fig. 2B).

The parasite was found by PCR analysis in 83 tissue samples of 90 samples and using digestion analysis in 55 tissue samples of the 90 samples taken from the infected animals in different periods after post-inoculation. The statistical comparison of the two approaches shows that 62.2% were positive by microscopic observation and 92.2% were positive by PCR, and were statistically similar (*P* > 0.05).

In this study, the agreement on the qualitative assessment was fair for digestion method (*n* = 6) and PCR assay (*n* = 55) (Table 2). Divergent results between techniques were observed in 29 samples: 28 were positive by PCR and negative by the microscopic observation analysis, while only 1 was positive by the microscopic analysis and negative by PCR. These divergent results were not statistically significant regarding to McNemar Chi-square test (*P* ˃ 0.05).

**Table 2:** Results of microscopic analyses and PCR for detection of *Toxocara cati* larvae in tissue samples of BALB/c mice sacrificed from day 1 to 28th after artificial infection

		PCR		
		Negative	Positive	Total
Digestion method analysis	Negative	6	28	34
	Positive	1	55	56
	Total	7	83	90

## Discussion

Although the life cycles of most helminths of zoonotic importance are well known, there are still major gaps in our knowledge especially in the fields of *Toxocara* species infection*.* In 1956, the life cycle of *T. cati* was first described by Dr. Sprent [22]. He argued that the life cycle of *T. cati* includes parthenic hosts that acquires infection by consuming larval in the tissues of the intermediate hosts. He also discussed the larvae were found in the liver, lungs and muscles after first week of infection, but did not reach the alimentary tract.

In the first part of the present study, by counting the number of larvae per gram of tissue, we have shown that toxocariasis caused by *T. cati* in BALB/c mice presents a dynamics that is very similar to the infection by this helminth or by *T. canis* in BALB/c mice [13, 23]. It has been shown that tissue digestion and conventional evaluation such as microscopic observations could remain a reliable tool for the diagnosis of toxocariasis in affected mice, given that they recovered large quantities of larvae during the course of the experimentally infection [24–28]. In BALB/c mice, orally infected with 2500 larval eggs, parasite larvae were detected for the first time in the liver at day 1 and the recovery time in the tissues continued until the day twenty-eighth. The number of larvae in the tissues decreased continuously from day 3 after infection so that no larvae recovered on day 28 after inoculation. Therefore, due to our data, it can be said that the acute phase of the disease occurred around day 7 and recovery between days 14 and 28 after inoculation. A similar kinetic has been described in male Wistar rats and Mongolian gerbil [5]. On day 14 of the experiment, the larvae were found in the lungs, heart and the kidneys and this coincided with their disappearance from the liver. These findings coincide with those of Prokopic and Figallova [29] who used a dose of 2500 T*. canis* embryonated eggs and Sprent [22] with a dose of 5000 T*. cati* eggs. The research results revealed that hatching of *T. cati* eggs in mouse intestines is faster than that of *T. canis* eggs. In a study using a dose of 2000 T*. cati* embryonated eggs, higher concentrations of larvae were found in the liver on the first day post-inoculation and in the lungs on days 2 and 3 post-inoculation. Also, in a similar survey by Hrckova et al. [30] using a dose of 1000 T*. cati* embryonated eggs and starting the study 4 h post-inoculation, recovered larvae in the liver as from 12 h post-inoculation and the maximum count was estimated on day 3 post-inoculation, the same as in the lungs.

One of our main goals was to compare the sensitivity of the conventional method of microscopic observation of the parasite with the PCR technique performed with specific primer. According to our previous study, primer species pairs that were able to detect parasite DNA in tissues were used [21]. The use of a special primer pair can increase the ability of PCR assays for parasite DNA. Regarding this issue, assessment of *T. cati* larvae were consistently positive from days 1 to 28 after experimental infection. Due to the higher sensitivity of PCR, and its comparison with conventional approaches to diagnosing infections, including helminth diseases [31], earlier detection of the parasite using PCR was expected. The very high infectious inoculation that has been used can be considered as the reason for the lack of significant differences in methods. This probably caused a large number of larvae to migrate simultaneously, which also reached microscopically detectable levels. However, it is important to note that *T. cati* is present in the host liver after 5 days of inoculation [5]. If migrating, their presence in tissue samples can be detected by PCR. The fact that PCR was positive throughout the recovery period while the microscopic method was only positive for inoculation at the beginning indicates that PCR assay is a better choice when the prevalence of infection or intensity of infection is low [32, 33]. Interestingly, the more intense parasite DNA amplification bands coincided with the highest amount of parasite larvae in the tissues. A similar correlation of parasite specific DNA and amount of parasite larvae was described using a PCR–RFLP to evaluate parasite in tissue samples of BALB/c mice infected with *T. canis* [33].

In the current study, the 600 bp band was derived from mice tissues DNA, which was confirmed by testing positive DNA samples. In the early stage of the infection, it was interesting not to observe the 600 bp band in some of the tissue samples, as larval DNAs were expected to be present in all specimens. A possible explanation for this finding could be a differential tendency of the genus primer pair for animals experimentally infected and parasite DNA. In this regard, it could be assume that this primer pair would be have a much higher affinity for *T. cati* larvae DNA than mice DNA. Undoubtedly, a visual inspection at the agarose gel documented in the figure confirms this possibility. For example, from lanes 1 to 4, very strong 600 bp bands are present. On the other hand, during later periods of infection, when the number of larvae is already decreasing, weaker 600 bp band is appears.

In the second part of the current study, we investigated if the tissue migration path of the larvae in BALB/c mice was similar to the observed in other rodents. We also tried to demonstrate that PCR performed with a genus-specific primer was more sensitive than traditional digestion and microscopic observation to detect larval migration in different tissues. The analysis of digested tissues and microscope examination showed that from the gastrointestinal luman site the larvae penetrated through circulation to the liver and then reached the lungs and other organs. This hepatic and pulmonary stage occurred within 24 h of infection.

Cardillo et al., [8] showed that *T. cati* causes considerable brain lesions in BALB/C on days 4 to 28 after inoculation and Zibaei et al., [5] reported through the study of brain tissue the importance of *T. cati* as causal agent of neurotoxocariasis. In addition, it has been shown recently *Toxocara* is included among one of the most common nematodal infections of the central nervous system [34]. This helminth is more commonly associated with seizure, dementia, or behaviour disorders mimicking primary psychiatric diseases [35]. One of the interesting aspects of the present study was the absence of larvae on microscopic examination during migration into brain tissue, while in the molecular assay a weakly band of 600 bp was detected in the brain tissue on the 28th day post-inoculation. Thus, we believe that the PCR we described will provide better performance in evaluating the detection of *T. cati* larvae in tissue samples from brain of infected mice.

## Limitations

The present study faced limitations such as resource constraints and lack of access to sequencing tests. Molecular testing with high sensitivity/specificity has been done to overcome some limitations. In addition, the migration pattern of *T. cati* larvae on carcasses and eyes will be investigated in the future studies.

## Conclusion

Finally, the results of the present study demonstrated that the experimental infection with *T. cati* in BALB/c mice presents the same kinetics as other rodents. This can be better characterized by PCR with a specific primer than by microscopic observation of larvae after tissue digestion. In addition, these findings also showed that simultaneous classical parasitological analysis and PCR with the genus primers were to most extent sensitive to detect parasites in different tissues.

## Supplementary Information

**Additionalfile 1:** **Supplementary Fig. 2. **Full-length gel images of PCR assay. (A) Representative results of polyacrylamidegel electrophoresis (1%) PCR products amplified from digested tissue samples ofinfected groups. The present study confirms that *Toxocara cati* larvaereached tissues from the alimentary canal. Molecular size marker 100kb (M) andlanes 1 to 4 indicate samples from the liver, lungs, heart, kidneys tissueswith DNA 600 bp. Lane 5 provide the presence a small amounts of DNA in the braintissue on the 28th day post-inoculation. (B) Result of polyacrylamide gelelectrophoresis of PCR products amplified from tissue samples of control group.The findings of the study showed that the tissues are free of *Toxocara *catilarvae. The negative control (NC) without DNA and positive control (PC) withstandard DNA 600 bp, lanes 1 to 5 represents tissue samples from the liver,lungs, heart, kidneys, and brain.

**Additional file 2**. STROBE Statement—Checklist of items that should be includedin reports of cross-sectional studies.

## Data Availability

All the data generated or analyzed in this study are included in this published article. The datasets used and/or analyzed during the current study are available from the corresponding author on reasonable request. More complete data and information are provided in the supplement file according to the STROBE checklist (Additional file 2).

## Abbreviations

Bp: Base pairs
CT: Covet toxocariasis
gDNA: Genomic DNA
NIH: National institutes of health
NT: Neurotoxocariasis
OLM: Ocular larva migrans
PCR: Polymerase chain reaction
RFLP: Restriction fragment length polymorphism
*T. **canis*: *Toxocara* *canis*
*T. **cati*: *Toxocara* *cati*
VLM: Visceral larva migrans
