# Supplementary material for: Larva migrans in BALB/c mice experimentally infected with Toxocara cati ensured by PCR assay
Source: BMC Vet Res. 2022 Jul 5;18:257. doi: 10.1186/s12917-022-03366-6 (PMC9254581; doi:10.1186/s12917-022-03366-6)
Supplement: Supplementary file 1 — Additionalfile 1: Supplementary Fig. 2. Full-length gel images of PCR assay. (A) Representative results of polyacrylamidegel electrophoresis (1%) PCR products amplified from digested tissue samples ofinfected groups. The present study confirms that Toxocara cati larvaereached tissues from the alimentary canal. Molecular size marker 100kb (M) andlanes 1 to 4 indicate samples from the liver, lungs, heart, kidneys tissueswith DNA 600 bp. Lane 5 provide the presence a small amounts of DNA in the braintissue on the 28th day post-inoculation. (B) Result of polyacrylamide gelelectrophoresis of PCR products amplified from tissue samples of control group.The findings of the study showed that the tissues are free of Toxocara catilarvae. The negative control (NC) without DNA and positive control (PC) withstandard DNA 600 bp, lanes 1 to 5 represents tissue samples from the liver,lungs, heart, kidneys, and brain. [file 12917_2022_3366_MOESM1_ESM.doc]

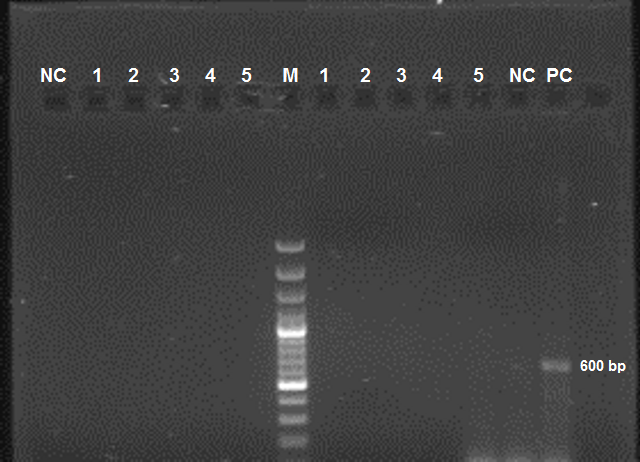


**
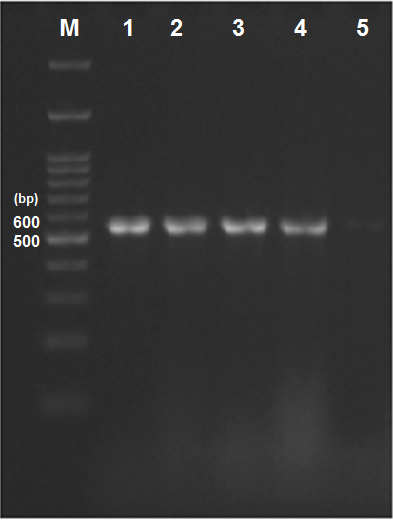
**

**Additional file 1**

Supplementary Fig. 2. Full-length gel images of PCR assay.

(A) Representative results of polyacrylamide gel electrophoresis (1%) PCR products amplified from digested tissue samples of infected groups. The present study confirms that *Toxocara cati* larvae reached tissues from the alimentary canal. Molecular size marker 100kb (M) and lanes 1 to 4 indicate samples from the liver, lungs, heart, kidneys tissues with DNA 600 bp. Lane 5 provide the presence a small amounts of DNA in the brain tissue on the 28th day post-inoculation. (B) Result of polyacrylamide gel electrophoresis of PCR products amplified from tissue samples of control group. The findings of the study showed that the tissues are free of *Toxocara* cati larvae. The negative control (NC) without DNA and positive control (PC) with standard DNA 600 bp, lanes 1 to 5 represents tissue samples from the liver, lungs, heart, kidneys, and brain.
