# Supplementary material for: Larva migrans in BALB/c mice experimentally infected with Toxocara cati ensured by PCR assay
Source: BMC Vet Res. 2022 Jul 5;18:257. doi: 10.1186/s12917-022-03366-6 (PMC9254581; doi:10.1186/s12917-022-03366-6)
Supplement: Supplementary file 2 — Additional file 2. STROBE Statement—Checklist of items that should be includedin reports of cross-sectional studies. [file 12917_2022_3366_MOESM2_ESM.doc]

STROBE Statement—Checklist of items that should be included in reports of ***cross-sectional studies***

|  | Item No | Recommendation |
| --- | --- | --- |
| **Title and abstract** | 1 | (*a*) Larva migrans in BALB/c mice experimentally infected with Toxocara cati ensured by PCR assay |
|  |  | (*b*) Background: Toxocara cati, the cat roundworm, is a parasitic nematode that known to cause toxocariasis in intermediate hosts and humans. In this study, we characterized the dynamics of T. cati larvae migration in BALB/c mice after inoculation with eggs and ensured the migration detecting the larval DNA by a PCR. To evaluate the dynamics of larval migration and distribution, twenty-four BALB/c mice were orally inoculated with 2500 T. cati infective eggs and the visceral organs of the infected animals were examined by pepsin digestion and microscopic parasite counts, followed by PCR at day 1 to 28 post infection.  Results: The PCR assays were successfully used for detection of T. cati larvae in tissue samples and T. cati larvae and the DNAs were found in the liver, lungs, heart, kidneys and the brain. We detected T. cati in 92.2% of tissue samples by PCR, 30% higher than the conventional pepsin digestion technique.  Conclusion: Although the sensitivity of microscopic larvae count and PCR to detect parasite’s presence in these tissues was almost statistically similar; however PCR showed higher sensitivity and our PCR assay may constitute a valuable alternative for the diagnosis of T. cati infections. |
| Introduction | | |
| Background/rationale | 2 | Toxocara cati, a global cosmopolitan and typically neglected parasitic zoonosis, is an ascarid nematode in the family Toxocaridae [1, 2]. Felids are the most common definitive hosts of T. cati, while humans as one of the many mammalian and many animals such as rodents and birds can serve as paratenic hosts [3]. Small mammals such as mice usually acquire the infection when they ingest infective Toxocara species eggs and are considered as a natural source of infection in the environment for predators [4, 5]. On the other hand, humans become infected by accidental ingestion of infective eggs from various environmental sources contaminated with definitive hosts feces. Toxocariasis encompasses four clinical syndromes: VLM, OLM, NT, and CT. Presentation and outcome of infection are determined by interaction between the host and the parasite [6].  Toxocara cati is a parasitic nematode of felids and BALB/c mice has been used as a model to study the host-parasite relationship in human and/or animal toxocariasis [7-9]. The larvae hatch and migrate out of the intestine to become lodged in organs and tissue [8]. Infection of rodents with T. cati has been carried out in different strains of rats and mice [5, 10]. However, the kinetic time-lines and migratory route have not been established in the BALB/c strain. The gold standard for the diagnosis of the majority of helminthic infections is still conventional microscopy and shown it is one of the most important diagnostic method to determine parasite migration routes and lesions caused by Toxocara species in paratenic hosts [5, 8, 11-15]  Polymerase chain reaction (PCR)-based techniques have revolutionized many areas of study including parasitic infection diagnosis. PCR is widely used mainly because its sensitivity allows the amplification of gDNA fragments from minute amounts of parasite material [16]. Performing a PCR assay does not require any parasitological proficiency and can be easily adapted by diagnostic laboratories that routinely use molecular assays to detect other pathogens. Several reports have shown a high PCR sensitivity for detection of Toxocara DNA in various biological samples from different hosts [17-19]. |
| Objectives | 3 | In the current study, the sensitivity between conventional methodology and PCR was compared to characterize T. cati infection in BALB/c mice. This comparison was carried out in two steps. First stage, initially was established the dynamics of infection by digestion method and microscopic observation, and PCR analysis of tissue samples. Then, the larvae migration route by PCR and parasitological examination was evaluated. |
| Methods | | |
| Study design | 4 | In this study, we characterized the dynamics of T. cati larvae migration in BALB/c mice after inoculation with eggs and ensured the migration detecting the larval DNA by a PCR. To evaluate the dynamics of larval migration and distribution, twenty-four BALB/c mice were orally inoculated with 2500 T. cati infective eggs and the visceral organs of the infected animals were examined by pepsin digestion and microscopic parasite counts, followed by PCR at day 1 to 28 post infection. |
| Setting | 5 | Worm samples were taken from cats with toxocara referred to Karaj veterinary clinics by taking anti-helminthic drugs; unembryonated eggs were harvested from adult female worms. Unembryonated T. cati eggs were collected from the faeces of naturally infected cats, incubated for three weeks at 25 °C for embryonation, following the technique described by Zibaei and Uga, [20]. Before inoculation, the eggs were washed three times with distilled water to remove formalin. |
| Participants | 6 | (*a*) Balb / c mice and cats with toxocariasis referred to clinics in Karaj. |
| Variables | 7 | Not applicable |
| Data sources/ measurement | 8* | Experimental design and protocol  Twenty-four animals were divided into 7 groups: control group (n = 6, with distilled water), six infected groups (n = 3/group). Mice were allowed to acclimatize for 1 week prior to commencing experiments. Total, 18 mice of infected groups were each orally inoculated with 2500 T. cati embryonated eggs suspended in 0.5 mL of saline solution, by stomach tube. The animals were monitored daily for clinical signs during routine animal care.  Recovery of larvae  Each 3 mice were anaesthetized with intraperitoneal injections of ketamine (60 mg/kg) and xylazine (8 mg/kg), then euthanized and necropsied on days 1, 3, 7, 14, 21, and 28 post-inoculation. Larvae were recovered and counted from the liver, lungs, heart, and kidneys using a pepsin digestion method and the modified Baermann technique [4]. The brain were removed individually and pressed between two slide-glasses to count the larvae under a light microscope. The controls were euthanized at the end of the study days.  Larval DNA  The animals groups (infected and control) were necropsied and their liver, lungs, heart, kidneys and brains were removed. Each organ was macerated separately and weighed in 5 fractions (quintuples) of 10 mg to 20 mg, for subsequent extraction of gDNA. All the quintuple samples of organs were frozen to -80 ºC for at least 24 h prior to initiating the DNA extraction procedure.  DNA isolation  Purify gDNA was isolated from samples using FavorprepTM Tissue Genomic DNA Extraction Mini Kit (Favorgen Biotech, Ping-Tung, Taiwan, and China) regarding the manufacturer’s instruction. The isolated DNA was eluted in 10 mM Tris–HCl and stored at -20 °C until further use.  PCR primers  A primer pair JW4 and NC2 (Forward primer 5′-ACTGTCGAGGATGAGCGTGA-3′ and Reverse primer 5′-TTAGTTTCTTTTCCTCCGCT-3′, respectively) was used to target a 600-bp fragment of the ribosomal ITS1 and ITS2 regions of T. cati [21].  PCR conditions  All PCR reactions were carried out in 25 μL final volume containing 12.5 μL of Super master mix 2X (Favorgen Biotech, Ping-Tung, Taiwan, China), 1.5 μL of each forward and reverse primer (10 pmol/μL), 3 μL of DNA template. The final reaction volume was adjusted to 25 μL with sterile demineralised water. The thermal profile of the PCR reaction was as follows: pre-denaturation at 94 °C for 5 min, followed by 35 cycles at 94 °C for 30 sec, annealing at 55 °C for 30 sec, extension at 72 °C for 60 sec, and a single final extension cycle at 72 °C for 7 min. After amplification, PCR products were electrophoresed on 1% agarose gel with 3 μg/mL GelRed, and a 100-bp ladder was used as DNA sizemarker for estimating the size of the amplicons and photographed using a gel documentation system (UV Transilluminator, QUANTUM SD4-1000, VILBER, France). |
| Bias | 9 | Describe any efforts to address potential sources of bias |
| Study size | 10 | Explain how the study size was arrived at |
| Quantitative variables | 11 | Explain how quantitative variables were handled in the analyses. If applicable, describe which groupings were chosen and why |
| Statistical methods | 12 | (*a*) Comparison of sensitivity between conventional and PCR techniques to detect larval infection was evaluated by the Chi-square test and the agreement between techniques, by the McNemar Test. The level of significance was set at 5% (P-values < 0.05). All data analyzing were using by SPSS 21.0 statistical software. |
|  |  | (*b*) Describe any methods used to examine subgroups and interactions |
|  |  | (*c*) Explain how missing data were addressed |
|  |  | (*d*) If applicable, describe analytical methods taking account of sampling strategy |
|  |  | (*e*) Describe any sensitivity analyses |
| Results | | |
| Participants | 13* | (a) Balb / c mice and cats with toxocariasis referred to clinics in Karaj |
|  |  | (b) Give reasons for non-participation at each stage |
|  |  | (c) Consider use of a flow diagram |
| Descriptive data | 14* | (a) Balb / c mice and cats with toxocariasis referred to clinics in Karaj |
|  |  | (b) Twenty-four animals were divided into 7 groups: control group (n = 6, with distilled water), six infected groups (n = 3/group) |
| Outcome data | 15* | The outcome of the infection was initially determined by counting the number of larvae that migrated to the tissue in the days after inoculation. The maximum number of tissues larvae was 40 (± 11.34) larvae observed on day 3 post-inoculation. From the 7th day onwards, the amount of recovered larvae steadily decreased until day 28.  Determination of infection by PCR depended upon the nucleotide sequence used as primers. Performing of PCR using the genus primer able to detect T. cati DNA over a far longer period from days 1 to 28, the last day evaluation. Interestingly, more intense parasite DNA amplification bands coincided with the highest amount of parasite larvae. |
| Main results | 16 | (*a*) Give unadjusted estimates and, if applicable, confounder-adjusted estimates and their precision (eg, 95% confidence interval). Make clear which confounders were adjusted for and why they were included |
|  |  | (*b*) Report category boundaries when continuous variables were categorized |
|  |  | (*c*) If relevant, consider translating estimates of relative risk into absolute risk for a meaningful time period |
| Other analyses | 17 | Report other analyses done—eg analyses of subgroups and interactions, and sensitivity analyses |
| Discussion | | |
| Key results | 18 | In the first part of the present study, by counting the number of larvae per gram of tissue, we have shown that toxocariasis caused by T. cati in BALB/c mice presents a dynamics that is very similar to the infection by this helminth or by T. canis in BALB/c mice [13, 22]. It has been shown that tissue digestion and conventional evaluation such as microscopic observations could remain a sufficient tool for the diagnosis of toxocariasis in affected mice, given that they recovered large quantities of larvae during the course of the experimentally infection |
| Limitations | 19 | The present study faced limitations such as resource constraints and lack of access to sequencing tests. Molecular testing with high sensitivity/specificity has been done to overcome some limitations. |
| Interpretation | 20 | Give a cautious overall interpretation of results considering objectives, limitations, multiplicity of analyses, results from similar studies, and other relevant evidence |
| Generalisability | 21 | Discuss the generalisability (external validity) of the study results |
| Other information | | |
| Funding | 22 | The authors state that they have not received any funding or grants to write and publish the results of this study. |

*Give information separately for exposed and unexposed groups.

**Note:** An Explanation and Elaboration article discusses each checklist item and gives methodological background and published examples of transparent reporting. The STROBE checklist is best used in conjunction with this article (freely available on the Web sites of PLoS Medicine at http://www.plosmedicine.org/, Annals of Internal Medicine at http://www.annals.org/, and Epidemiology at http://www.epidem.com/). Information on the STROBE Initiative is available at www.strobe-statement.org.
